# Emotional Creativity as Predictor of Intrinsic Motivation and Academic Engagement in University Students: The Mediating Role of Positive Emotions

**DOI:** 10.3389/fpsyg.2016.01243

**Published:** 2016-08-25

**Authors:** Xavier Oriol, Alberto Amutio, Michelle Mendoza, Silvia Da Costa, Rafael Miranda

**Affiliations:** ^1^Department of Management and Public Policies, Universidad de Santiago de ChileSantiago, Chile; ^2^Department of Social Psychology and Methodology of the Behavioral Sciences, Faculty of Psychology, University of the Basque Country/Euskal Herriko UnibertsitateaDonostia-San Sebastian, Spain; ^3^Faculty of Education, Universidad Autónoma de ChileTemuco, Chile; ^4^Department of Social Psychology and Methodology of the Behavioral Sciences, University of the Basque Country/Euskal Herriko UnibertsitateaDonostia-San Sebastian, Spain; ^5^Departamento de Psicología, Escuela de Gobierno y Políticas Públicas, Pontificia Universidad Católica del PerúLima, Perú

**Keywords:** dispositional emotional creativity, class-related emotions, academic engagement, intrinsic motivation, university students

## Abstract

**Objective:** Emotional creativity (EC) implies experiencing a complex emotional life, which is becoming increasingly necessary in societies that demand innovation and constant changes. This research studies the relation of EC as a dispositional trait with intrinsic motivation (IM) and academic engagement (AE).

**Methods:** A sample of 428 university Chilean students, 36.5% men and 63.5% women, with ages from 18 to 45 years-old (*M* = 20.37; *DT* = 2.71). Additionally, the mediating function of class-related positive emotions in this relation is explored.

**Results:** The obtained data indicate that developing high levels of dispositional EC enhances the activation of positive emotions, such as gratitude, love and hope, in the classroom. Furthermore, EC predicts IM and AE of university students by the experience of positive emotions.

**Conclusion:** These results compel us to be aware of the importance that university students can understand the complexity of the emotional processes they undergo. A greater control of these emotions would allow students to maintain higher levels of interest in their studies at the different educational stages and to avoid the risk of school failure.

## Introduction

Based on a socio-constructivist perspective, Averill (1980, 2005) understands creativity as a structure associated with emotion, in which emotions are the result of objective and subjective creative efforts made by the individual. For Averill (2005, 2009), emotional creativity (EC) is a dispositional trait consisting in experiencing a complex emotional life, which depends largely on the social norms that give coherence to the experienced emotions. Thus, Averill considers that it is possible to foster the development of creativity from early ages.

Today, the impact of EC in the academic level bears particular relevance as we take into account the fundamental role that emotions play in the teaching-learning processes (Pekrun and Perry, 2014; Amutio et al., 2015). There is an increasing tendency to study the influence of emotions on creative processes and, inversely, the impact of creative ideas or products on the generation of emotions (St-Louis and Vallerand, 2015). However, dispositional EC implies, according to Averill (2005), an even greater complexity in terms of emotional processes. This complexity might be crucial to improve students’ satisfaction and intrinsic interest in their own learning process.

Emotional creativity is defined as the ability to experience and express original, appropriate, and authentic combinations of emotions. Hence, a person with high EC will experience emotions that are more complex (Averill and Thomas-Knowles, 1991). Averill (2005) believes that this kind of individuals spend more time on recognizing emotions and that they have a preparedness for this. In a different way, emotional intelligence is characterized by the individual’s ability to identify, understand and express, regulate and use their own emotions and the emotions of others (Salovey and Mayer, 1990; Bisquerra and Pérez-Escoda, 2007; Peña-Sarrionandia et al., 2015). According to this perspective, the processes of emotional regulation may favor an improvement of thought and enhance creative processes (Gross, 2013; Medrano et al., 2013). However, people with high EC do not need to perform these regulatory processes because they know how to generate their own personalized combinations of emotions. Thus, they create original emotional reactions that benefit creativity (Ivcevic et al., 2007). In sum, the principal difference between EC and emotional intelligence lies in that people with high EC distance themselves from the common reactions to generate original emotional reactions. Furthermore, they can find inspiration in negative affectivity, i.e., becoming inspired and excited when writing about these experiences (Ivcevic et al., 2007; Averill, 2009).

According to Averill’s (1980, 2009) tenets, individuals with high EC possess the capacity of being more sensitive to the experienced emotions and devote more time to recognize them, which would arouse these people’s enthusiasm for generating a novel emotional reaction. Averill (2009, 2013) defined four dimensions of EC: (1) *Novelty*, which represents the acquisition of new knowledge from former behaviors; (2) *Effectiveness*, implying that to be creative a response has to be of potential use for the person or the group; (3) *Authenticity*, as a creative response that constitutes a reflection of individual values and beliefs of the world and an authentic expression of them, and not merely a copy of others expectations; (4) *Preparedness*, which implies that years of preparation are required before achieving creativity within a specific area.

To assess EC, Averill (1999) created the *Emotional Creativity Inventory (ECI)*, a unique 30 items self-perception instrument that evaluates the ability to experience and express emotions in a novel, effective, and authentic way. High scores in the ECI have been related to various personality dimensions, including *Openness to Experience* and *Agreeableness*, but not to Neuroticism, Extraversion, or Conscientiousness. In a closer relation with learning processes, EC is seen as a predictor of the development of creative writing and artistic activities (Ivcevic et al., 2007; Averill, 2009) and has been also been related to cognitive (Averill, 2005; Fuchs et al., 2007) and artistic creativity (Barron and Harrington, 1981; Averill and Thomas-Knowles, 1991; Feist and Barron, 2003). Additionally, people that show high levels of EC would be more likely to enjoy new emotional experiences and learning in addition to higher levels of flow during regular activities (Averill and Nunley, 2010).

A variety of emotional states influences the learning processes, in both the motivational phase, when students are weighing whether to commit or not and to which goal, and the volitional phase, when students are reaffirming their commitment to the selected goal (Järvenoja and Järvelä, 2005; Lüftenegger et al., 2016). Emotions experienced in the classroom affect the performance of students, as well as their interest, commitment and personality development, which, in turn, affects the social climate in the classrooms and educational institutions (Pekrun, 2006; Amutio et al., 2015; López-González and Oriol, 2016). Academic emotions comprise different actors, such as teachers, students, parents, and school employers. They may also vary according to the different learning moments as, for example, when doing homework, house chores, during class or when taking tests or exams (Pekrun, 2000; Pekrun and Perry, 2014).

When academic activities generate satisfaction, happiness, hope or pride, students feel more motivated before a task, pay more attention, and show greater self-control of their own learning process, feel more academically engaged, and tend to make more academic efforts (Roffman, 2004; Davey et al., 2005; Knoop, 2011; Oriol et al., 2016). On the contrary, experiencing negative emotions may cause bad academic adaptation, that is, students can feel bored or develop a feeling of frustration that can lead to school failure (Pekrun et al., 2004; Ruthig et al., 2008; López and Calderon, 2011; D’Mello and Graesser, 2012). Therefore, the experienced emotions are especially relevant to the disaffection or commitment students may feel regarding their own learning process (Skinner et al., 2008; Pekrun and Linnenbrink-García, 2012).

The interest that arouses as an immediate reaction to a new task is an affective state that involves feelings of excitement, concentration and attention and, thus, is a fundamental variable for motivation and commitment to learning. Consequently, the activation of emotions in the classroom is directly related to the perception and behavior of the students in relation to the academic tasks (Pekrun, 2006; Knoop, 2011). Despite all these findings, research on collective or group emotions in the classroom is still scarce (Aritzeta et al., 2015).

Motivation and engagement are studied complementarily, since the literature considers them as key variables in the learning process and for the improvement of academic results (Woolfolk and Margetts, 2007; Parra, 2010; Ainley and Ainley, 2011). Self-determination theory distinguishes between extrinsic and intrinsic motivation (IM) (Ryan and Deci, 2000; Deci and Ryan, 2008; Vallerand et al., 2008). IM correlates positively with academic engagement (AE) (Ryan and Deci, 2009), academic satisfaction (Miquelon et al., 2005; Ratelle et al., 2007), intrinsic interest (Vallerand et al., 1993, 2008), and enjoyment (Black and Deci, 2000; Álvarez et al., 2009). Unlike extrinsically motivated students, those intrinsically motivated tend to be more creative and to acquire knowledge better, because they engage more and voluntarily devote more time and energy to study (León et al., 2015).

Academic engagement has been conceptualized as the extent to which students are committed to school and motivated to learn (González et al., 2015). The engagement concept has been traditionally applied in the work area, and is understood in terms of task behavior, effort, persistence, participation and work habits (Schaufeli et al., 2002a,b; Kahu, 2013). However, in the last years, this construct has become increasingly complex with the advent of new instruments that define AE in terms of three dimensions: *behavioral* (i.e., time on task), *emotional* (i.e., interest and value), and *cognitive* engagement (i.e., self-regulation and learning strategies; Fredricks et al., 2004; González et al., 2015). Recent research has suggested a fourth dimension of student’s engagement in school, namely personal agency, which conceptualizes students as proactive, as agents of action by showing initiative, interest and making suggestions (Veiga et al., 2015). Moreover, AE has been researched from different approaches, focusing on a diversity of topics related to students, including task value, class participation, satisfaction, emotional and academic involvement, achievement, motivation, perceived control and self-efficacy, self-esteem, emotional intelligence, and well-being (Caballero et al., 2007; Appleton et al., 2008; Ros et al., 2012; González et al., 2015; Oriol et al., 2016).

De la Fuente et al. (2010) underline that AE is a positive state of high energy, dedication and fullness during the execution of academic tasks, and it is characterized by favorable and lasting IM toward those tasks. Furthermore, engagement is considered a mediator between motivation and performance (Salanova et al., 2010; González et al., 2015) and is regarded as a vital factor for social-personal development and school success. More generally, positive psychology is interested in psychological adjustment, and considers students’ engagement as a major factor of it (Seligman and Csikszentmihalyi, 2000; Fernández-Zabala et al., 2015).

To summarize, one of the key aspects in psychology studies on the educational area over the last few years has been the incorporation of emotions and their connection with the cognitive processes related to learning (Linnenbrink-Garcia and Pekrun, 2011). Emotions play a fundamental role in students’ interest in learning as well as in their commitment to the accomplishment of educational tasks. Hence, a deeper understanding of the antecedents that generate the activation of a different set of emotions in the learning process and how they influence the variables related to perseverance and performance is necessary (Pekrun and Perry, 2014).

Emotions are associated with the way in which students perceive learning and may influence both their motivation and their AE, whether inhibiting or promoting the achievement of their goals (Turner et al., 2002; Rozendaal et al., 2005; Amutio et al., 2015). Therefore, people with high EC would develop academic curiosity because of their need to innovate and generate new knowledge (Averill, 2005). In this sense, it is considered that higher levels of EC as a dispositional trait will promote the activation of academic emotions within the classroom and this, in turn, will generate higher levels of IM for studies and AE.

### Aims of the Present Study

One of the greatest challenges for the teaching in higher education is to enable students to be more autonomous and to show a firm commitment to constant learning. Therefore, the general aim of this study is to prove the existing relationship between emotional EC and its dimensions, and college students’ motivation and engagement to learning.

Based on the cited research, the hypotheses of this study are: (1) EC and its dimensions: *Preparedness*, *Novelty* and *Effectiveness/Authenticity*, will be (positively) related to the activation of positive emotions in university students. (2) EC and its dimensions will predict AE and IM. (3) Positive emotions will mediate the relation between EC and AE. (4) Positive emotions will also mediate the relation between EC and IM. To prove the hypotheses, different multiple mediation models will be tested controlling for the variables of sex and age.

## Materials and Methods

### Participants and Procedure

The sample is composed of students from three Chilean universities: *Universidad Autónoma de Chile*, in Talca (25.5%), and in Santiago (36.7%), *Universidad de Talca* (33.2%), and *Universidad Católica del Maule* (14.6%). In total, the sample was made up of 428 university students, 36.5% men and 63.5% women with ages from 18 to 45 years (*M* = 20.37; *DT* = 2.71).

The sample was selected by convenience, taking into account the ease of access to it by the researchers. Permission from the competent authorities was requested in each university for conducting the study. Approval was obtained by the Committee of Ethics of the corresponding universities, authorizing the students to participate in the study. The registered data was alphanumerically coded, ensuring confidentiality and anonymity, in order to comply with the Personal Data Protection Act by the Ethics Committee for Research related to Human Beings (CEISH).

Prior to the beginning of the study, students were also given an informed consent that explained the study’s objectives and stated the confidentially of the gathered data, which would only be used with research purposes.

The instruments were administered in the classroom where students usually attended classes in approximately 25–35 min.

### Measures

All the instruments were applied in the classrooms of the respective universities during the last week of the semester, after final examinations.

- *Shortened version of Emotional Creativity Inventory* (*ECI*-S). This scale evaluates dispositional personal traits (Averill, 1999). The Spanish version of the ECI-S (Soroa et al., 2015) is a self-report questionnaire composed of 17 items and three dimensions, which are: (1) *Preparedness* or emotional disposition, which is the capacity of understand and learn about ones’ own and others’ emotions (e.g., “I think about my emotions and try to understand my emotional reactions”). (2) *Novelty*, or the capacity or ability of experiencing new or unusual emotions (e.g., “I have felt a mix of emotions that probably other people have never felt”). Finally, (3) *Effectiveness/Authenticity*, which refers to the capacity or ability of expressing emotions, which, in the end, translates into benefits for the individual or group, directly and honestly (e.g., “the way I live and express my emotions helps me in my relationships with others”). This is a Likert-type scale that ranges from 1 = *totally disagree* to 6 = *totally agree.* In the present sample, reliability was satisfactory for EC, with a total alpha of 0.85, and for the three dimensions *Preparedness* (α = 0.80), *Novelty* (α = 0.84), and *Effectiveness/Authenticity* (α = 0.88). Finally, a CFA was conducted on the scale considering a three-factor solution and the fit was acceptable: χ^2^ = 244.40, *p* < 0.000; χ^2^*/gl*. = 4.4; NFI = 0.92; CFI = 0.92; IFI = 0.92; RMSEA = 0.05; SRMR = 0.04.
- *Positivity test* (Fredrickson, 2009). This test evaluates 10 adjectives that describe each positive emotion at different intensity levels (amusement, compassion, gratitude, hope, joy, interest, inspiration, love, pride, and surprise) in a Likert-type scale in which 0 = *nothing* and 4 = *a lot.* Students were asked to respond this scale considering how often they experienced or not those emotions in class during the semester. As for reliability, α was 0.83. However, each emotion was used independently in this study.
- *Academic engagement*. The brief version (nine items) of *Utrech’s Work Engagement Scale* (UWESS-9), elaborated by Schaufeli and Bakker (2003), and adapted and validated in Chile by Parra and Pérez (2010) for students (UWES-S), was applied. The scale presents nine statements of vigor, absorption and dedication before studies. An example item is: “I feel strong and vigorous when I’m studying or going to class.” The student should respond based on frequency of occurrence of these items over time by selecting one out of the six Likert-type alternatives, i.e., (0 = *Never*, 1 = *A few times a year*, 2 = *Once a month or less*, 3 = *A few times a month*, 4 = *Once a week*, 5 = *A few times a week*, 6 = *Every day*). In this study, the reliability of this scale was 0.89. The CFA show an acceptable fit χ^2^ = 26.35, *p* < 0.000; χ^2^*/g.l*. = 1.7; NFI = 0.98; CFI = 0.98; IFI = 0.98; RMSEA = 0.04; SRMR = 0.01.
- *Intrinsic motivation.* The *CEVEAPEU* questionnaire is an instrument to assess the learning strategies of university students elaborated and validated by Gargallo et al. (2009). This scale contains 141 items and 15 dimensions, but only the dimension related to IM was used for this study. This dimension includes three items related to the IM university students feel for their studies (e.g., “I feel satisfied when I understand the contents deeply”). For this work, this dimension showed a reliability of 0.83. Regarding CFA, a unique factor solution was also tested and the results were acceptable, χ^2^ = 2.799, *p* = 2.47; χ^2^*/g.l*. = 1.4; NFI = 0.98; CFI = 0.98; IFI = 0.98; RMSEA = 0.03; SRMR = 0.01.

### Design and Analyses

This is a cross-sectional study based on different scales applied to university students. Statistical descriptions and bivariate correlations were calculated through the SPSS 20.0. CFA was performed using AMOS. To analyze the effects of positive and negative emotions and EC (multiple mediation) in relation to AE and IM, the bootstrap procedure proposed by Preacher and Hayes (2008) was applied through the SPSS macro MEDIATE for models with multiple independent variables. This analysis estimates the indirect effect, standard errors and confidence intervals. The non-parametric bootstrapping procedure was used with 5000 repetitions to calculate the 95% confidence intervals. The indirect effect is significant if the confidence interval does not exceed zero value (Preacher and Hayes, 2008; Hayes, 2013). To test the indirect effects and calculate the effect size, we used the PROCESS macro that calculated the ratio of the indirect effect to the total effect (Hayes, 2013). Sex and age were used as covariates.

## Results

### Descriptive and Correlation Analyses

Descriptive statistics of the different variables being studied (**Table 1**) show a medium-high experience of positive emotions, along with the correlation indices of the different variables, which are direct, positive and significant in the expected sense. In fact, it can be noted that EC correlates significantly with IM and AE, while significant correlations were also obtained with *Gratitude*, *Hope*, and *Love*.

**Table 1:** Mean and standard deviation for emotional creativity (EC), intrinsic motivation (IM), academic engagement (AE) and positive emotions and correlations between the main variables.

Predicted variables	Mean and SD	*Emotional creativity*		*Preparedness*		*Novelty*		*Effectiveness*	
					
		*M*	*(SD)*	*M*	*(SD)*	*M*	*(SD)*	*M*	*(SD)*
		4.00	(0.67)	4.64	(0.89)	3.59	(0.97)	4.28	(0.92)
*IM*	(*M* = 4.99; *SD* = 0.911)	0.299^∗∗^		0.409^∗∗^		0.100		0.309^∗∗^	
*AE*	(*M* = 3.83; *SD* = 1.05)	0.172^∗∗^		0.313^∗∗^		-0.034		0.289^∗∗^	
(1) Amusement	(*M* = 2.63; *SD* = 0.80)	0.108		-0.016		0.088		0.129^∗^	
(2) Surprise	(*M* = 2.26; *SD* = 0.89)	0.122		0.029		0.092		0.125	
(3) Gratitude	(*M* = 2.77; *SD* = 0.91)	0.202^∗∗^		0.126		0.097		0.242^∗∗^	
(4) Hope	(*M* = 2.73; *SD* = 0.81)	0.181^∗∗^		0.186^∗∗^		0.018		0.271^∗∗^	
(5) Inspiration	(*M* = 2.64; *SD* = 0.84)	0.104		0.197^∗∗^		-0.029		0.193^∗∗^	
(6) Interest	(*M* = 2.57; *SD* = 0.78)	0.075		0.163^∗^		0.011		0.060	
(7) Joy	(*M* = 2.91; *SD* = 0.75)	0.040		0.022		-0.032		0.126	
(8) Love	(*M* = 2.71; *SD* = -0.86)	0.207^∗∗^		0.111		0.122		0.190^∗∗^	
(9) Pride	(*M* = 2.64; *SD* = 0.94)	0.101		0.134^∗^		-0.044		0.236^∗∗^	
(10) Compassion	(*M* = 2.27; *SD* = 0.85)	0.083		0.025		0.040		0.120	


*^∗^*p* < 0.05; ^∗∗^*p* < 0.01.*

### Mediation Analyses

We use SPSS macros for indirect effect bootstrapping (Hayes, 2013), which provide indicators for indirect effects, multiple mediations, standard errors, and confidence intervals derived from bootstrap distribution. An indirect effect is significant if the confidence interval does not include the value 0. According to the previous analyses, those emotions that showed a significant relation with the indirect variable (EC) were entered as possible mediators in the tested models. In total, seven multiple mediation models were tested taking EC and its dimensions as indirect variables, AE and IM as direct variables, and controlling for sex and age, which were used as covariates.

**FIGURE 1:**
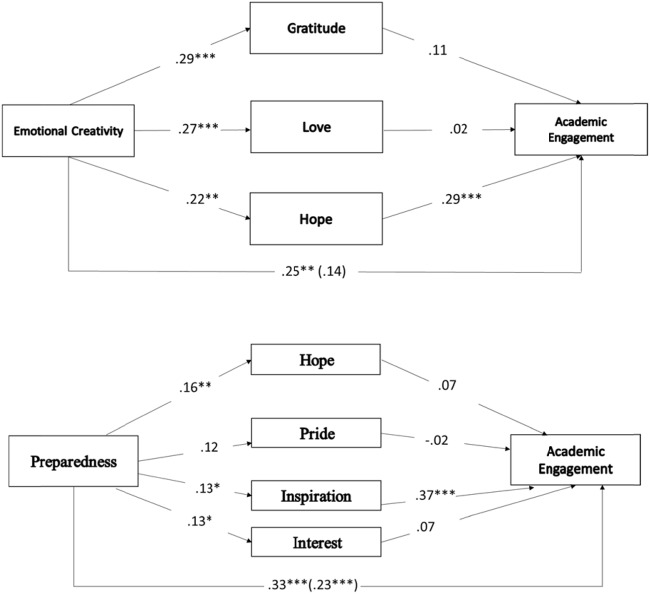
**(A)** Multiple mediation model of the association between Emotional Creativity and Academic Engagement via class-related emotions. Coefficients are unstandardized. **(B)** A multiple mediation model of the association between preparedness and AE via class-related emotion. Coefficients are unstandardized. ^∗^*p* < 0.05; ^∗∗^*p* < 0.01; ^∗∗∗^*p* < 0.001.

The total and direct effects on EC and AE are shown in **Figure 1**. The total effect of EC on AE (*B* = 0.25, *SE* = 0.10, CI [0.057, 0.454]) decreases after entering the mediators, producing a total mediation (*B* = 0.14, *SE* = 0.10, CI [-0.051, 0.343]). In addition, as seen in **Table 2**, EC has an indirect effect on AE through the variable *hope*. In regards to the *preparedness* dimension, there is a significant total effect on AE (*B* = 0.33, *SE* = 0.07, CI [0.185, 0.477]), which decreases after entering the mediators (*B* = 0.23, *SE* = 0.07, CI [0.096, 0.372]), producing partial mediation. In this case, an indirect effect of preparedness on AE through the variable *inspiration* is observed. Finally, a significant total effect can be seen in the relation between *Effectiveness/Authenticity* and AE (*B* = 0.29, *SE* = 0.07, CI [0.116, 0.412]), which decreases when mediators are included (*B* = 0.15, *SE* = 0.06, CI [0.000, 0.267]), as well as an indirect effect of this dimension of creativity (*Effectiveness/Authenticity)* on AE through *Inspiration* (see **Table 2**).

**Table 2:** Significant indirect effects of EC on AE and IM.

VI on VD	Mediator	Parameter estimate	*SE*	Lower 95% BC CI	Upper 95% BC CI
EC on AE	Hope	0.065^∗^	0.03	0.012	0.153
Preparedness on AE	Inspiration	0.051^∗^	0.02	0.073	0.123
Effectiveness on AE	Inspiration	0.059^∗^	0.02	0.015	0.134

EC on IM	Hope	0.039^∗^	0.02	0.009	0.119
Preparedness on IM	Interest	0.035^∗^	0.01	0.006	0.084
Effectiveness on IM	Inspiration	0.0059^∗^	0.02	0.015	0.0123


*BC, bias-corrected; CIs, confidence intervals. ^∗^*p* < 0.05.*

**FIGURE 2:**
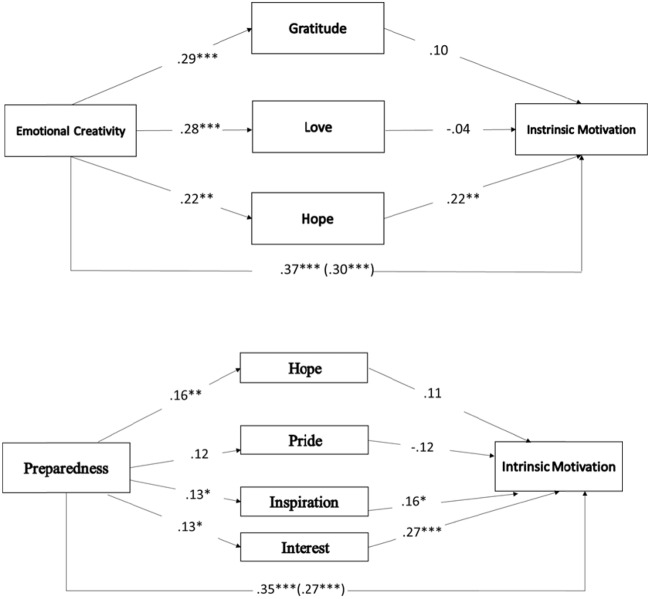
**(A)** A multiple mediation model of the association between EC and intrinsic motivation via class-related emotions. Coefficients are unstandardized. **(B)** A multiple mediation model of the association between Preparedness, and IM via class-related emotions. Coefficients are unstandardized. ^∗^*p* < 0.05; ^∗∗^*p* < 0.01; ^∗∗∗^*p* < 0.001.

Regarding the results of the mediations conducted between EC and IM, a total effect (*B* = 0.37, *SE* = 0.08, CI [0.206, 0.538]) which decreases after including the mediators (*B* = 0.30, *SE* = 0.08, CI [0.137, 0.473]) is observed (see **Figure 2**). As shown in **Figure 2**, out of the three emotions entered as mediators, *hope* is the only one that shows a significant indirect effect. In the *preparedness* dimension, the total effect between this variable and IM is also significant (*B* = 0.35, *SE* = 0.06, CI [0.231, 0.478]). The same is true for the indirect effect, although this decreases due to the effect of the mediators (*B* = 0.27, *SE* = 0.05, CI [0.154, 0.387]) and resulting in partial mediation. In addition, in this case, there is an indirect effect through the emotion of *interest*. Lastly, the total effects of *Effectiveness/Authenticity* on IM also presented significant results (*B* = 0.26, *SE* = 0.06, CI [0.115, 0.234]). When entering the emotions as mediators, the effect decreased, yet still being significant (*B* = 0.17, *SE* = 0.06, CI [0.135, 0.311]). Finally, an indirect effect through *inspiration* is seen in this last dimension (see **Figure 2** and **Table 2**).

## Discussion

The obtained results provided support for our hypotheses. In the first hypothesis, EC and its dimensions were expected to be related to the positive emotions experienced in the classroom. The results showed that *EC* is associated with the positive emotions of *gratitude*, *hope*, and *love*. Regarding its dimensions, *Preparedness* is positively related to *hope, pride, inspiration*, and *interest.* The second dimension, *Effectiveness/Authenticity*, shows a significant positive relation with *amusement*, *gratitude*, *hope*, *inspiration*, *love*, and *pride*. Finally, the third dimension of *Novelty* did not show any positive association.

Emotions such as *hope, inspiration* and *interest*, which have been related to creativity and its dimensions in this study, are associated with the capacity of being committed and motivated, and of displaying personal competences to change negative circumstances (Tugade and Fredrickson, 2004; Fredrickson, 2009; López and Calderon, 2011). Thus, EC would imply a deployment of complex emotional resources that would enhance the students’ coping capacity to the extent that they are able to develop a positive perception of in-class experiences and reduce the experience of negative emotions. Other emotions that appear related to EC in this study, such as *gratitude* and *love*, are more associated with affective experiences involving other persons. In this sense, Averill (2005) emphasizes that EC implies investing time in recognizing other people’s emotions, along with being able to express ones’ own emotions, facilitating the exchange of emotional experiences in the classroom.

As expected in the second hypothesis, EC and its dimensions were predictors for both IM and AE. These results appear especially relevant if we consider the importance of these two variables to increase academic performance (Woolfolk and Margetts, 2007; De la Fuente et al., 2010; Amutio et al., 2015; López-González et al., 2016). Specifically, college students more instrínsecally motivated show a deeper processing and learning, higher levels of AE and increased performance (Fenollar et al., 2007; Richardson et al., 2012; Moreno-Murcia and Silveira, 2015).

It is also noted that *preparedness*, which would imply the capacity of a person to understand his/her own emotions and be willing to explore them, is the dimension more strongly associated with IM and AE. Students with high levels of *Preparedness* would be more willing to explore new emotions and, thus, would have a better understanding of their emotional processes. This, in turn, would make them perceive the academic tasks as more challenging and motivating (Deci and Ryan, 2014). The results show that EC, understood as the capacity of experiencing complex emotions, allows students to enjoy the learning processes, even to the extent of transforming negative emotional states into inspiration forms (Ivcevic et al., 2007), showing greater commitment to the tasks.

The emotion of *hope* shows a significant mediating effect on the relation between EC and IM, and with AE. *Hope* can emerge before negative or uncertain situations and is associated with a tendency to feel full of energy and inspired to plan some positive actions in order to change negative circumstances (Tugade and Fredrickson, 2004; Fredrickson, 2009). According to our results, EC predicts the activation of hope in the classroom. In turn, experiencing this emotion stimulates motivation and engagement. This result is consistent with other studies’ (López and Calderon, 2011). In addition, *hope* is linked to academic achievement. Specifically, hopeful beginning college students have a higher overall GPA. Hopeful students are energetic and full of life. They see the future better than the present and are able to develop many strategies to reach goals as well as to plan contingencies in the event of facing problems along the way. As such, obstacles are viewed as challenges to overcome. High-hope students focus on success and experience greater positive affect and less distress. On the contrary, low-hope students may lack the energy to get things done and experience high anxiety and lowered self-confidence and self-esteem.

In the *Preparedness* dimension it can be observed that the emotion of *inspiration* mediates the relation between EC with AE and IM, while *interest* mediates the relation with IM. *Interest* fosters the feeling of having opportunities of learning something new and *inspiration* enhances transcending the usual and the routine through the perception of new possibilities (Watson, 2000; Fredrickson, 2009). For its part, *inspiration* also mediates the relation between the *Effectiveness/Authenticity* dimension of EC, and IM, and AE.

The results provide support for hypotheses 3 and 4. Recent studies have shown that positive academic emotions are the forerunners of AE, since they promote satisfaction with the activities related to learning in university students (Knoop, 2011; Linnenbrink-Garcia and Pekrun, 2011; Oriol et al., 2016), and this study confirms that experienced emotions act as mediators in the relation between EC with IM and AE. According to Averill’s (1980, 2005) socio-constructivist tenets, increasing the levels of EC during early educational stages becomes fundamental to allow students to experience firm commitment to their studies and, thus, enhancing their autonomous learning. EC implies experiencing a complex emotional life and activating positive emotions in the classroom to face the challenges derived from the complexity of the current educational systems. The lack of congruence between the contents taught and the real interests of students requires reassessing the learning processes in such a way that the new curriculum awakens true interest and generates real satisfaction in students. The development of EC may be one of the greatest challenges for the educational systems to achieve the student’s reconnection with their own learning processes and reduce the risk of school failure. To reach this goal, launching social-emotional learning programs (Coelho et al., 2014) and even, programs directly aiming at boosting the experience of positive emotions, like mindfulness programs, for both students and teachers, is crucial (Franco et al., 2014; Amutio et al., 2015; López-González and Oriol, 2016; López-González et al., 2016). Activating positive emotions will allow students to perceive themselves as successful in the tasks execution (self-efficacy), as opposed to negative emotions, which are related to more perceptions of failure (Amutio et al., 2015; Oriol et al., 2016; Tze et al., 2016).

## Conclusion

The results confirm the need to change teaching methodologies developed at university level to promote IM patterns and higher levels of AE. The constant changes produced in today’s society make students loose attention and interest quickly if they are exposed to monotonous activities that do not generate novel emotional experiences. Therefore, activities and tasks conducted by professors must connect with everyday experiences of students in order to activate emotions that help them generate greater meaning to their knowledge. Tasks that involve creative processes are much more attractive, produce greater emotional arousal, greater autonomy and promote self-construction of knowledge. Consequently, activities that are new and surprising and capable of generating rich emotions that stimulate learning and facilitate management of emotional situations are recommended in higher education (e.g., simulations and case studies where students need to face future situations in their respective fields of work) in order to optimize the AE of university students.

As for the limitations and future orientations, it should be noted that research on academic emotions is becoming highly complex. Evaluating specifically these emotions during situations in which students are submitted to different pressure levels and academic requirements (i.e., exams period) becomes necessary. This study in particular focused on the positive emotions that are experienced in the classroom, but it might be replicated in other moments or circumstances of the learning process. Second, the concept of AE comprises behavioral, emotional and cognitive components (Fredricks et al., 2004; González et al., 2015). Nevertheless, this work used a scale that contained the vigor, dedication and absorption dimensions adapted from an engagement construct that has been applied in the work area. Taking into account the complexity of this construct, deeper research on the relation between EC and AE should be carried out using other instruments.

## Author Contributions

XO: data analyses, results, and discussion; Universidad de Santiago de Chile. AA: introduction, results, and discussion; University of the Basque Country (UPV/EHU). MM: data analyses; Universidad Autónoma de Chile. SC: data analyses; University of the Basque Country (UPV/EHU). RM: review of the literature; Universidad Católica del Perú.

## Conflict of Interest Statement

The authors declare that the research was conducted in the absence of any commercial or financial relationships that could be construed as a potential conflict of interest.
